# An outbreak investigation of visceral leishmaniasis among residents of Dharan town, eastern Nepal, evidence for urban transmission of *Leishmania donovani*

**DOI:** 10.1186/1471-2334-13-21

**Published:** 2013-01-18

**Authors:** Surendra Uranw, Epco Hasker, Lalita Roy, Filip Meheus, Murari Lal Das, Narayan Raj Bhattarai, Suman Rijal, Marleen Boelaert

**Affiliations:** 1 B.P. Koirala Institute of Health Sciences, Ghopa, 56700, Dharan, Nepal; 2 Department of Public Health, Institute of Tropical Medicine, Antwerp, Belgium

**Keywords:** Kala-azar, Visceral leishmaniasis, Risk factor, Outbreak, Disease transmission, Sand fly infection, Nepal

## Abstract

**Background:**

Visceral leishmaniasis (VL) is a predominantly rural disease, common in the low lands of eastern Nepal. Since 1997 VL cases have also been reported among residents of the city of Dharan. Our main research objective was to find out whether there had been local transmission of VL inside the city.

**Methods:**

We conducted an outbreak investigation including a case–control study; cases were all urban residents treated for VL between 2000 and 2008 at BP Koirala Institute of Health Sciences, a university hospital in the city. For each case, we selected four random controls, with no history of previous VL; frequency-matched for age. Cases and controls were subjected to a structured interview on the main exposures of interest and potential confounders; a binominal multilevel model was used to analyze the data. We also collected entomological data from all neighborhoods of the city.

**Results:**

We enrolled 115 VL patients and 448 controls. Cases were strongly clustered, 70% residing in 3 out of 19 neighborhoods. We found a strong association with socio-economic status, the poorest being most at risk. Housing was a risk factor independent from socio-economic status, most at risk were those living in thatched houses without windows. ‘Sleeping upstairs’ and ‘sleeping on a bed’ were strongly protective, OR of 0.08 and 0.25 respectively; proximity to a case was a strong risk factor (OR 3.79). Sand flies were captured in all neighborhoods; in collections from several neighborhoods presence of *L*. *donovani* could be demonstrated by PCR.

**Conclusion:**

The evidence found in this study is consistent with transmission of anthroponotic VL within the city. The vector *P*. *argentipes* and the parasite *L*. *donovani* have both been identified inside the town. These findings are highly relevant for policy makers; in VL endemic areas appropriate surveillance and disease control measures must be adopted not only in rural areas but in urban areas as well.

## Background

Visceral leishmaniasis (VL), is a vector-borne parasitic disease, common in Nepal’s plains known as *Terai* lowlands. Kala-azar (KA), the name used for VL in South Asia, is a significant public health problem in the country. Twelve districts in the eastern and central *Terai* regions, bordering the Indian state of Bihar, are endemic. In the Indian subcontinent, VL is mainly caused by *Leishmania donovani* and its transmission is assumed anthroponotic through the bite of the sand fly species *Phlebotomus argentipes*[1]. Nepal’s border with the highly endemic state of Bihar, home to 85% of India’s VL cases, makes Nepal a key partner in the regional VL elimination program [2].

VL is known as a disease that predominantly affects the rural poor [3-7]. Outbreaks have been reported in several rural villages [8] but VL is also spreading into areas that were previously non-endemic [9,10]. Since 1997 VL cases have also been reported among urban residents in the town of Dharan. Many of those were treated at B.P. Koirala Institute of Health Sciences (BPKIHS), the only hospital facility in the town. A survey conducted in July 2006 showed an average VL incidence rate of 2.05% per year (for 2004–2006) in certain neighbourhoods of Dharan [11]. Dharan is located on the foothills of the Mahabharata range, at the ecological transition zone between the sub-tropical forest of the plains and the mountains. The residents of Dharan town use this forest as their source of firewood and fodder. The historic town centre is surrounded by suburban residential areas of recent legal settlers as well as slum areas, where displaced people have moved in at high speed over the past ten years - during the civil conflict- seeking safety and job opportunities. Those recent immigrants could of course have been infected with *L*. *donovani* outside Dharan town, but there is a definite concern about the possibility of local transmission of *L*. *donovani* in the urban/suburban area for several reasons. The sand fly vector was observed in peripheral neighbourhoods of Dharan town in previous studies [12,13]. Dharan town is also known to host a community of intravenous-drug users (IDUs), which might act as a catalyser for VL transmission. The HIV infection rate in IDUs in Nepal is estimated at 14-39%, compared to approximately 0.5% in the general population [14,15]. Sharing of needles by IDUs could shift the mode of transmission of VL [16] and was the basis for previous outbreaks in Europe [17].

To study the possibility of urban transmission of VL in Dharan town we conducted a case–control study and collected entomological data from all neighbourhoods of the town.

## Methods

### Study area and population

**Figure 1:**
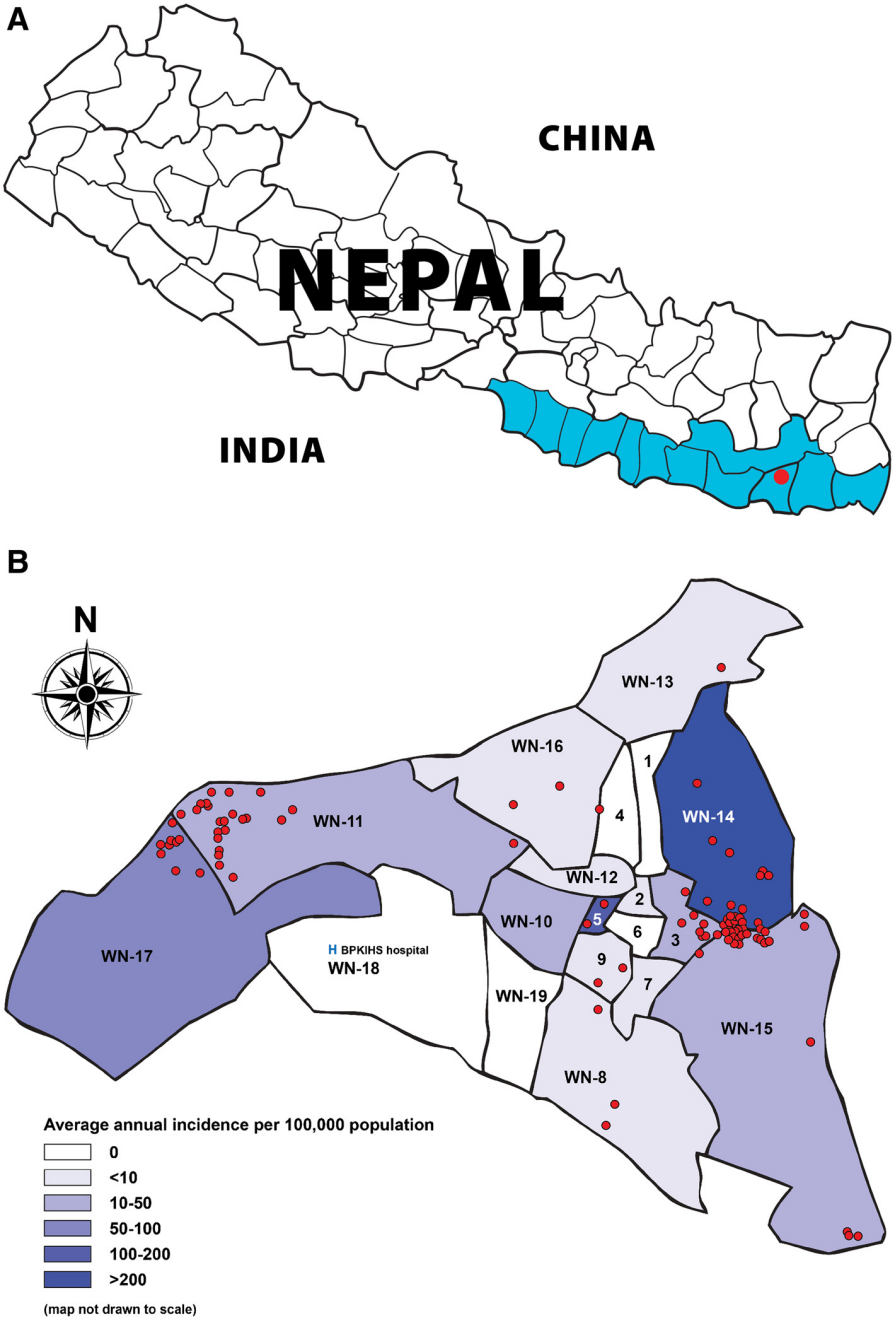
**A Visceral leishmaniasis endemic area of Nepal (highlighted), study site ‘Dharan town’ (red dot), eastern Nepal. B Distribution of visceral leishmaniasis cases (red dots) by ward in Dharan town, eastern Nepal (2000–2008).**

Dharan town is located in Sunsari district in the eastern region of Nepal. The town is administratively divided into 19 “*wards*” (see Figure 1B) covering a surface area of 21.12 km^2^ with a total population of 95,332 (Census 2001). The literacy rate is about 72%, higher than the national average of 40%. Dharan is a town of recent growth through migration. These migrants earn their living from small scale business and sales of agricultural produce or depend on daily manual labor for their livelihood. They usually squat on unoccupied land, living in makeshift houses. BPKIHS Dharan, a 700-beded tertiary care hospital is located in the southwest of the town, with a reference VL treatment centre since 1997. 

### Study design and case definitions

This study was designed as an outbreak investigation including a case–control study; we selected four controls for each case. For ethical reasons, we included only individuals aged above 5 years. All study subjects had been residents of Dharan town for at least 2 years at time of diagnosis (cases) or at time of enrolment (controls). Cases (fever ≥ 2 weeks duration with clinical splenomegaly) were those with parasitologically confirmed VL registered at BPKIHS between January 2000 and December 2008 were enrolled in the study. Controls were individuals randomly selected from the town population who had not suffered from VL in the past and did not present with fever and/or splenomegaly on the day of survey. Controls were frequency-matched for age to cases.

BPKIHS drains a substantial proportion of VL cases from the eastern region and all cases from Dharan. We exploited the patient database of BPKIHS to identify and recruit VL cases. This patient register, which has clinical and epidemiological information of all admitted VL patients since 1997, is well maintained and has been monitored by external clinical monitors on a regular basis as several patient cohorts were entered into clinical studies [18-20].

### Sample size

Based on the reported numbers of VL cases, we expected to be able to enrol at least 110 cases. In that case a ratio of 4 controls for each case would be sufficient to detect an odds ratio of 2 with a power of 80% at a significance level of 5% for a factor to which 15% of controls are exposed.

### Data collection

The study was conducted between September and November 2009. We collected clinical and epidemiological information from the BPKIHS hospital patient records and through interviews. We compiled the list of all VL patients who gave ‘Dharan municipality’ as their residence at the time of admission. We then traced those persons at their homes. Controls were selected randomly from the updated 2007/8 voting register of Dharan municipality using a random-number table. This register was based on a census of all citizens carried out in 2007/8 in preparation of the elections and included all recent settlers at that time. All households are listed in the voting register by ward and household number, name and age of family members’ ≥ 16 years is enumerated. To allow for inclusion of the children in the group of controls in the same proportion as cases, we replaced the randomly selected adult voter by a child in 1 out of every 6 controls. In such case instead of interviewing the adult voter, we enrolled randomly one of all the under 16 years old present in that household. If there were no children in the household sampled, a child was selected from the house of the nearest neighbor.

After obtaining individual written informed consent, the field workers conducted structured interviews. The questionnaire contains detailed questions on individual and household level variables. Individual-level variables included among others sex, marital status, literacy, employment status, intravenous drug use, migration status and regular forest visit. Household variables included among others proximity to previous VL cases, socioeconomic status and housing characteristics. Houses were subdivided into ‘thatched houses without windows’ ‘thatched houses with windows’ and ‘brick houses’. Cases were asked to report their status vis-à-vis the different variables at the time of their illness; for controls the status at time of interview was recorded. In case of children, we interviewed parents or guardians.

Responses with regard to actual household income were unreliable. Therefore, socio-economic status was assessed for the household, based on a previously validated asset index [7]. Included in the asset index are ownership of: radio (s), CD player (s), television (s), video or DVD player (s), mobile phone (s), non-mobile phone (s), refrigerator (s), iron (s), sewing machine (s), watch (s), chair(s), sofa(s), table(s), cupboard(s), bicycle(s), motorcycle or scooter (s), and animal draw cart (s). Also included was type of toilet facilities, fuel used for cooking, number of sleeping rooms and availability of electricity. The asset index was converted into assets scores, using principal component analysis. Based on the assets scores, households were divided into four socio-economic layers.

All households enrolled were georeferenced, so were the boundaries of the study area. A map was created using Arcview GIS 9.2 (ESRI, Redlands, USA). We created an individual 50 meter radius buffer around each VL case household; any control living within any of those buffer zones was considered to be contact of a case. A case was considered as a contact if at any time before the diagnosis another case had occurred in the same house or in another house within a 50 meter radius.

Ethical approval for the study was obtained from the Institutional Ethical Review Board of the B.P. Koirala Institute of Health Sciences, Dharan, Nepal. The use of patient’s medical records in this study was part of the approved study protocol.

### Entomology

We captured sand flies in 10 randomly selected households in each of the 19 wards, thus a total of 190 houses in Dharan town were sampled. Collections took place at the 19 locations; one day in each location from October 3–23, 2009 during the second annual peak for *P*. *argentipes* density [21]. Sand flies were captured overnight using CDC light traps in each household, supplemented by mouth aspiration for 15 minutes the following morning to catch resting sand flies [22]. This was performed by trained insect collectors and supervised by an entomologist. After collection, flies were brought to the entomology laboratory at BPKIHS and examined under a binocular dissecting microscope; female *P*. *argentipes* were morphologically identified and separated from other insects. Female *P*. *argentipes* were pooled by household in a cryotube with 80% alcohol.

We applied polymerase chain reaction (PCR) technique as described by Bhattarai [12]. The molecular analysis was done by extracting DNA from sandflies by using the QIAmp DNA minikit (Qiagen, Germany), as per the manufacturer’s instruction. Firstly, a sand fly specific cytochrome b PCR was applied to verify the quality of the DNA present in each pool. Secondly, *Leishmania* specific PCR was used to detect *Leishmania infection*. The PCR positive samples were further sequenced in 3730 DNA Analyzer (Applied Bio systems, Foster City, CA, USA) in order to confirm that the amplified DNA corresponded well to the *Leishmania* donovani species.

### Statistical analysis

Data was double entered in a Microsoft Access database independently by two data entry clerks and the two files were compared after the completion of data entry. In case of discrepancies, corrections were made after reviewing the original questionnaire forms.

For data analysis, we used binominal multilevel model in Stata /IC V10.1 (Stata Corp., College Station, Tx, USA) with ‘ward’ (neighborhood) as random effect. Observed associations were assessed through multilevel logistic regression. All variables with a *P*-value ≤ 0.10 in univariate analysis were included in the multivariate logistic regression model. Variables for the final model were selected using the backward elimination strategy. The probability of removal was set at *P* = 0.05. We tested for biologically plausible interactions between factors retained. To test for interactions between ward and various factors, we fitted random slopes for all factors retained and used likelihood ratio testing to assess whether the model with random slope fits the data significantly better than the model without; probability for removal was set at 0.05.

## Results

**Figure 2:**
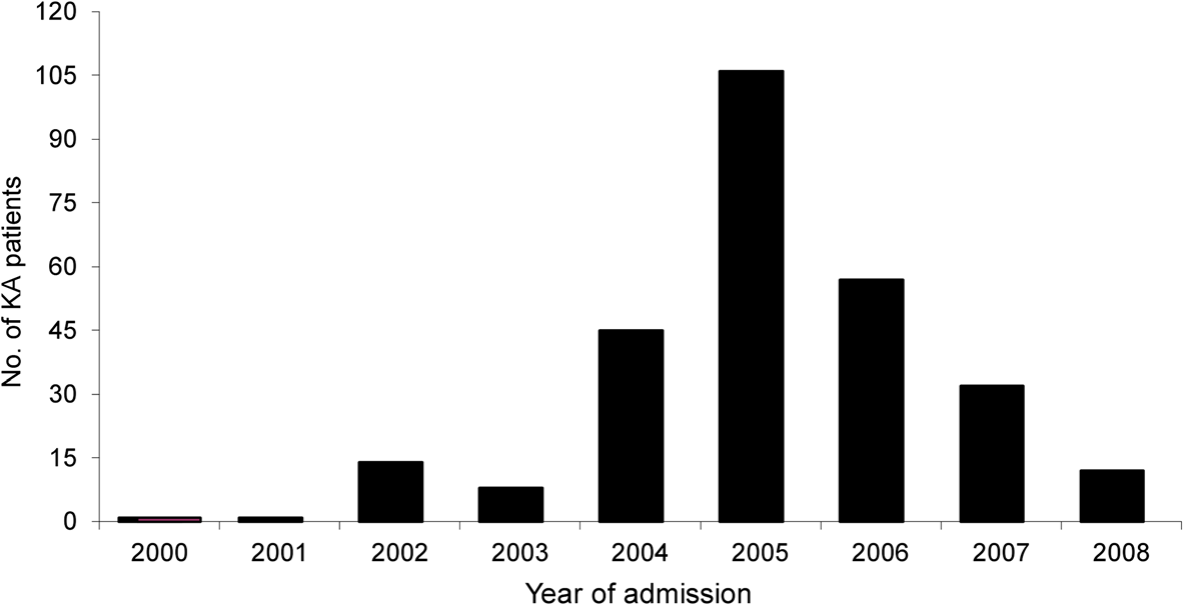
Number of visceral leishmaniasis patients admitted at BPKIHS hospital by year from Dharan town, Nepal, 2000–2008 (n = 276).

Between 2000 and 2008, BPKIHS admitted 276 VL cases from Dharan town with a peak in 2005 (Figure 2). We found a very focal distribution of VL, reflected in an intra-class correlation by neighbourhood (ward) of 50.5% (Figure 1B). Overall 70% of cases were resident in 3 out of 19 wards, particularly the peripheral wards of the town. We managed to trace 158 out of 276 VL cases at their home; there were 46 households with more than one VL case. Sampling only the most recent case from multiple case households we enrolled 115 VL cases and 448 controls in the study; 66 cases were male (57.4%) and 49 female (42.6%). The median age was 31, interquartile range (IQR) 18–42 years (Table 1). 

**Table 1:** Characteristics of visceral leishmaniasis cases and controls

**Factor**	**Cases(115)**	**Controls(448)**	**OR †(95%CI) ‡**	***P***-***value***
	**no. (%)**	**no. (%)**		
Age (year)				
Median (Inter quartile range)	31 (18 – 42)	36 (26 – 50)		
Sex				
Male	66 (57.4)	285 (63.6)	0.65 (0.40 – 1.05)	0.08
Female	49 (42.6)	163 (36.4)		
Marital status				
Married	69 (60.0)	325 (72.5)	0.55 (0.34 – 0.91)	0.02
Unmarried	46 (40.0)	123 (27.5)		
Religion group				
Hindu	99 (86.1)	403 (89.9)	0.78 (0.38 – 1.59)	0.50
Others (Budhist, Christian, Muslim)	16 (13.9)	45 (10.01)		
Literate				
Yes	101 (87.8)	407 (90.8)	0.65 (0.31 – 1.41)	0.28
No	14 (12.2)	41 (9.2)		

† OR, odds ratio; ‡CI, confidence interval.

Children ≤ 5 year old are excluded.

Table 2 shows the results of the ‘univariate’ risk factor analysis with ‘ward’ as random effect. Factors associa-ted with increased VL odds were: ‘history of migration’(OR 1.75, 95% CI 1.08 – 2.83), ‘living within 50 meters of a previous VL case’ (OR 6.60, 95% CI 3.69 – 11.82), ‘regularly visiting the forest’ (OR 6.08, 95% CI 3.49 - 10.61), ‘occupation as daily laborer’ (OR 2.99, 95% CI 1.78 – 5.02), and ‘living in a large-size household’ (OR 2.39, 95% CI 1.46 – 3.93). With thatched houses without windows as referent, we found odds ratios of 0.28 (95% CI 0.13-0.60) and 0.05 (95% CI 0.02-0.12) for those living in thatched houses with windows and in brick houses respectively. Sleeping upstairs (OR 0.03, 95% CI 0.01 – 0.21) and sleeping on a bed rather than on the floor (OR 0.09, 95% CI 0.03 – 0.24) were strongly protective, so was ‘ownership of cattle’ (OR 0.09, 95% CI 0.01-0.82). The risk for those reporting to ‘ever use a bednet’ was similar to that of those not using a bednet (OR 1.06, 95% CI 0.62 - 1.81). The bed nets in use were commercially produced and were not treated with insecticide. IV drug use was a risk factor (OR 2.3) but the association was not statistically significant (*p* = 0.17). 

**Table 2:** Factors associated with VL in a ‘univariate’ model with ‘ward’ as random effect

**Factors**	**No**. **exposed**		**OR †**	**95%CI ‡**	***P- *value**
	Case (%)*	Control (%)*			
	**(n = 115)**	**(n = 448)**			
Adult	95 (82.61)	393 (87.72)	0.68	0.35 – 1.31	0.25
Male sex	66 (57.4)	285 (63.6)	0.65	0.40 – 1.05	0.08
Married	69 (60.0)	325 (72.5)	0.55	0.34 – 0.91	0.02
Hindu religion	99 (86.1)	403 (89.9)	0.78	0.38 – 1.59	0.50
Literate	101 (87.8)	407 (90.9)	0.65	0.31 – 1.41	0.28
Large households (> 5 person)	54 (46.9)	125 (27.9)	2.39	1.46 – 3.93	0.001
Intravenous Drug use	6 (5.2)	13 (2.9)	2.30	0.69 – 7.66	0.17
Proximity to other VL cases within 50 m	52 (45.2)	32 (7.1)	6.60	3.69 – 11.82	<0.0001
Regular forest visit	59 (51.3)	67 (14.9)	6.08	3.49 – 10.61	<0.0001
Daily wage earner (vs fixed employment)	84 (73.0)	240 (53.6)	2.99	1.78 – 5.02	<0.0001
History of migration	66 (57.4)	214 (47.8)	1.75	1.08 – 2.83	0.02
Bed net use ever	83 (73.5)	330 (73.7)	1.06	0.62 – 1.81	0.81
Sleeping on a bed (vs on the floor)	94 (81.7)	439 (97.9)	0.09	0.03 – 0.24	<0.0001
Sleeping upstairs	1 (0.9)	121 (27.0)	0.03	0.01 – 0.21	0.001
Goat ownership	26 (22.6)	75 (16.7)	1.23	0.70 – 2.19	0.48
Cattle ownership	1 (0.9)	26 (5.8)	0.09	0.01 – 0.82	0.03
Pig ownership	34 (29.6)	149 (33.3)	0.86	0.52 – 1.44	0.58
Chickens/ducks ownership	14 (12.2)	76 (16.9)	0.62	0.31 – 1.22	0.16
Type of house					
Thatched house, no windows	33 (28.7)	21 (4.7)	Referent		
Thatched house with windows	62 (53.9)	170 (37.9)	0.28	0.13 – 0.60	0.001
Brick house	20 (17.4)	257 (57.4)	0.05	0.02 – 0.12	<0.0001
Socio economic status (by asset score level)					
Level 1 (poorest)	87 (75.7)	112 (25.0)	Referent		
Level 2	20 (17.4)	112 (25.0)	0.22	0.11 - 0.42	<0.0001
Level 3	7 (6.1)	112 (25.0)	0.09	0.03 - 0.23	<0.0001
Level 4 (least poor)	1 (0.9)	112 (25.0)	0.01	0.001 - 0.08	<0.0001

* Proportion (%) unless specified otherwise.

† OR, odds ratio; ‡CI, confidence interval with ‘ward’ as random effect.

The asset-index clearly distinguishes between the poorest (1^st^ quartile) from the richest (4^th^ quartile). Of all cases, 76% belonged to the poorest quartile and only 1% to the richest quartile of the study population. With the poorest quartile as referent we found odds ratios of 0.22, 0.09 and 0.01 for the second poorest, the second richest and the richest quartile respectively.

The multivariate model that fits the data best is a model with a random intercept for ‘ward’ and ‘proximity to a previous case’, ‘regular forest visits’, large size household’, ‘type of house’, ‘daily wage earner’, ‘sleeping upstairs’, ‘sleeping on a bed’ and ‘asset-index’ as independent variables. ‘Adult’ was kept in the model because cases and controls were matched on this factor. After we thus controlled for potential confounding by socio-economic status and other factors, ‘proximity to previous VL case’ (OR 3.79, 95% CI 1.86 – 7.71), ‘large size household’ (OR 3.55, 95% CI 1.84 – 6.83), and ‘regular forest visits’ (OR 4.25, 95% CI 2.15 – 8.40) remained strong and statistically significant risk factors for VL. Type of housing also remained strongly associated, with odds ratios of 0.36, (95% CI 0.15 – 0.84) for ‘thatched houses with windows and 0.26 (95% CI 0.09 – 0.73) for brick houses, both with thatched houses without windows as referent. Sleeping upstairs (OR 0.08, 95% CI 0.01 – 0.64), and sleeping on a bed rather than on the floor (OR 0.25, 95% CI 0.08 – 0.78) remained strongly and significantly protective. The association with socio-economic status remained very strong and there was a clear dose–response effect. The odds for VL consistently decreased as the level of socio-economic status increased (OR 0.44, 0.26, 0.04 for increase from quartile 1 to 2, 3, 4 respectively). Details are presented in Table 3. 

**Table 3:** Factors associated with VL in a ‘multivariate’ model with ‘ward’ as random effect

**Factors**	**OR †**	**95%CI ‡**	***P- *value**
Proximity to other VL cases ( within 50 m)	3.79	1.86 – 7.71	<0.0001
Regular forest visit	4.25	2.15 – 8.40	<0.0001
Large households size (>5 person)	3.55	1.84 – 6.83	<0.0001
Sleeping on a bed (vs on the floor)	0.25	0.08 – 0.78	0.016
Sleeping upstairs	0.08	0.01 – 0.64	0.017
Adult	1.07	0.46 – 2.46	0.88
Type of house			
Thatched house, no windows	Referent	-	
Thatched house with windows	0.36	0.15 – 0.84	0.019
Brick house	0.26	0.09 – 0.73	0.010
Socio economic status (by asset score level)			
Level 1 (poorest)	Referent	-	
Level 2	0.44	0.20 – 0.95	0.036
Level 3	0.26	0.08 – 0.81	0.020
Level 4 (least poor)	0.04	0.004 – 0.38	0.005

†OR, odds ratio; ‡CI, confidence interval.

Altogether 592 sandflies (female *P*. *argentipes*) were captured from 121 out of 190 households in Dharan town. These sand flies were pooled by household, resulting in 121 pools each containing 1–22 female *Phlebotomus argentipes*. All pools were found to be PCR positive for the *cytochrome b* gene, indicating the presence of good quality of DNA in these samples. Out of these 121 pools, 13 were found to be positive for *L*. *donovani* and originated from 6 wards (1, 5, 15, 16, 18 & 19); none of them was from the two worst affected wards (14 and 17). An important observation we made during our study is the inadequate coverage (≤ 70%) of the insecticide residual spraying (IRS), with exception of the three wards worst affected with VL (14, 15 & 17) where IRS coverage was ≥ 90%.

## Discussion

This is the first study to describe urban transmission of VL in Nepal, VL still being largely considered a disease of the rural poor. VL in Dharan town has a strongly clustered distribution reflected in an intra-class correlation for ward of 50.5%; worst affected were the more peripheral wards (Figure 1B). These are wards with new settlements of recent migrants where the poorest segments of the population reside and which lack adequate infrastructure, sanitation and housing. They are typically a rural–urban interface as most residents depend on daily manual labor for their livelihood (unpublished data), and therefore VL cases might have been easily exposed to *L*. *donovani* infection elsewhere. However the results of our study are more consistent with local urban transmission. We demonstrated the presence of *Phlebotomine* sand flies in all the Dharan wards as was observed in earlier studies [12,13] and we were able to confirm infection with *L*. *donovani* in some of the flies captured.

The final multilevel model showed a very strong association between VL and certain housing factors, those living in a thatched houses without windows having 3–4 times higher odds of kala-azar. Sleeping on a bed and upstairs were protective (OR 0.25, 95% CI 0.08-0.78 and OR 0.08, 95% CI 0.01 - 0.64 respectively). Proximity to previous VL cases was a strong risk factor for VL (OR 3.79, 95% CI 1.86 – 7.71). These associations are all consistent with local transmission. Regular forest visits was also associated though, with an odds ratio of 4.25 (95% CI 2.15 – 8.40), which would point at transmission outside the city.

The entomological data provide further evidence in support of local transmission of VL inside the city. *P*. *argentipes* sand flies were captured in all 19 wards of the town; in 13 out of 121 pools of sand flies captured, the presence of *L*. *donovani* infection could be confirmed. The fact that no *L*. *donovani* infected sand flies were captured in the two wards worst affected (wards 14 and 17) could be explained by the low yield of entomological sampling in these wards linked to recent Indoor Residual Spraying (IRS) in response to the VL outbreak. The 13 *L*. *donovani* positive pools did originate from 6 neighboring wards where IRS coverage had been substantially lower (wards 1, 5, 16, 18 & 19, IRS coverage 10-70%), however the absolute numbers of infected sandflies captured were low and random variation may have played a role. Since our study was retrospective, entomology data reflect the situation in 2009, rather than the situation at the time the VL cases were diagnosed. Our findings suggest that IRS has been highly effective in reducing vector abundance. To prevent further outbreaks of VL a scaling up to fully cover all wards could be recommended.

This study has one major limitation, it was a retrospective study looking back at cases occurring over a period of more than 10 years. Cases occurred from 2000 to 2008, but cases and controls were interviewed in 2009. For cases we asked for the conditions as they were at the time of their illness, for controls this was not possible so their answers reflect conditions in 2009. Conditions may have changed over this period and there is likely to be a recall bias among some of the cases interviewed; the entomological data also reflect the situation in 2009. We did perform a sensitivity analysis including only cases from 2005 onward but did not find any major differences in results (data not shown). Despite the limitations mentioned we did find strong indications in favor of urban transmission of anthroponotic VL. This may have important implications for VL control not only for Dharan but also for other cities in VL endemic regions on the Indian subcontinent.

A spatial analysis conducted in 1,744 cases reported from 1991 to 2000 in the city of Teresina, Brazil showed the highest incidence rate of VL in neighbourhoods with a rural–urban interface, located in the city’s peripheral areas and occupied by low-income populations [23]. A rapid disorganized increase in population associated with an increase in the number of VL cases in the city was also observed in another study [24]. The establishment of new residential colonies in the town and an increased migration from known endemic areas were also identified as the source of VL transmission into virgin areas in Shimla, India [25].

## Conclusion

With a very high likelihood the outbreak of VL observed in Dharan can be attributed to transmission within the town. The vector *P*. *argentipes* and the parasite *L*. *donovani* have been identified inside the town. Vector control activities (IRS) appear to have been effective in controlling the outbreak. However the vector is still present in all wards, in particular in those wards that did not have a very high IRS coverage. Ensuring adequate IRS coverage in all wards of the town is recommended. These findings also have implications for other urban areas on the Indian subcontinent. In endemic areas, surveillance for VL needs to be in place not just in rural areas but in cities as well.

## Competing interests

The authors declare that they have no competing interests.

## Authors’ contributions

SU, EH, FM, SR and MB designed the study, drafted the first version of the manuscript and finalised the manuscript. SU carried out the field survey. LR and MLD carried out the entomological survey and laboratory work. NRB performed the sand fly analysis. SU, EH and FM performed the statistical analysis . SU, EH, LR, FM, NR, SR and MB interpreted the results and finalised the manuscript. All the authors read and approved the final manuscript. MB is guarantor of the paper.

## Pre-publication history

The pre-publication history for this paper can be accessed here:

http://www.biomedcentral.com/1471-2334/13/21/prepub
